# Authority and the Medical Student

**Published:** 1910-09-03

**Authors:** 


### AUTHORITY AND THE MEDICAL STUDENT.

BY A MEDICAL HERETIC.

At the entrance of the medical school where he is destined to work for five years the student finds that he is face to face with Authority, spelt with not only a capital A, but in capitals right through. That is an inevitable complication which it is as useless to cavil at as it is to complain of the examination system. Discipline in a medical school must be maintained just as much as in any other institution, and no one in his senses will presume to question the desirability of prohibiting the professional student from making his own interests paramount and subserving to it those of the school and hospital to which he is attached. But there is another and entirely different kind of authority with which the tyro, if he be not wholly brainless nor wholly without independent power of reasoning, will encounter not only in his school-days, but throughout his professional career. It is the authority of personality, of words, theories, postulates, phrases, names, events—of a multitude of things to which he will be asked to bow the head in submissive acquiescence on pain of being considered a professional heretic, or, at the best, a professional nonconformist. He will meet it from the first, when he begins to dissect the dogfish and is given a teleological explanation of a structure that puzzles him, and it will follow him to his death, when his colleague signs his death certificate in accordance with the authoritative tautological classification of disease. There is a tendency on the part of the medical student to be ultra-orthodox. It needs an effort to break away from this kind of authority, to be a protestant, and to divine the truth Mohammed taught, that "every sect has a certain tract of heaven." Nor is it to be lightly deprecated, this unwavering faith in symbols, in theories, in words, and above all in persons, that inspires the young practitioner who has not yet attained his professional majority. All too soon, when he leaves the sheltering walls of his medical school, will he have to bear the brunt of carking opposition and contemptuous prejudice—as hateful as his own if he only knew it. The sneer at the homœopathist that his lecturer on pharmacology made, and that he laughed at—although he knew less of Hahnemann than of the great Hermann—the smile at the sentimentalism of the anti-vivisectionist, at the folly of the Eddyist, at the unwisdom of the anti-vaccinationist—all these in after-time will afford him scope for reflection and thought. When he presses shoulder against shoulder in that vast throng of sixty thousand odd competitors he finds that his neighbour, though professionally a heretic, is a good fellow, and, what is infinitely more powerful as a liberalising argument, a successful man. There are other measures of self-respect, says Emerson, for a man than the number of clean shirts he puts on in a day, and our friend the private patient judges men by an entirely different standard than do our equally good chums the examiners at the final. When we have discovered this amazing impudence in the man or woman who asks for our assistance, this horrible craving to be attended by a man, and not by an animated text-book, we are in a position to realise the truth of de Vigny's remark, *L'espérance est la plus grande de nos folies.*

For we leave our house appointments, our hospital schools, with a glow of hope. How excellently, and yet how pathetically, has the great explorer-doctor summed up the situation in one of his charming letters to Felkin. The passage is well known, but it will bear repetition, and I offer no apology for giving it in full: "Well do I remember the day when I received the magic title of Doctor of Medicine, and thought that then the whole world was my own. May far happier days and greater success be yours than have ever been granted me! But may I give you a word of warning? Keep yourself well in hand, and do not follow without very just cause the too modern developments of medicine. A sick man is no subject, but a feeling and suffering being whose sensibility is greatly heightened by pain. Be to your patients, in the first place, friend—then doctor. Our mission is a high and holy one, and the murmured thanks of a poor man far higher value than a couple of guineas; the knowledge that you have helped to save a sick child a far more beautiful reward than can ever follow a brilliant but risky operation, or the humbug of so-called scientific medicine. Do not laugh at my words. I have grown old and grey in the battle of life, but it is just this idealism that has helped me over many a bitter hour."

And it is just this idealism that we risk the losing of while we are walking the wards blindly subservient to authority that is founded on premises as dubious as those which buttressed the claims of any escobaritic defender of a *quodlibet* question. A professional man, especially in a profession such as ours, where only the initiated can judge of the merits of his case, must be made of very stern stuff if he dares to be a Daniel while in the company of his colleagues. It is true, medicine shows brilliant exemplars to those that have minds to follow the thorny road
Which leads through toil and hate to Fame's serene abode, and when in the distant future a medical Foxe arises (let us sincerely hope more truthful and more historic than his notorious prototype), he will find plenty of material at hand to justify the premise that the severest course of professional cramming has not always sufficed to etiolate and kill the plumule of idealism which sprouts in every young man's mind when he commences his life's work. The safeguarding factor in medical as in every other study is broad-mindedness—let me add with a knowledge of the principles of logic and a sense of humour. That magnificent passage in "Queen of the Air," where Ruskin speaks of the narrow-mindedness of religious bigots, is applicable with much truth and justice to medical bigotry. Yet no man can be a bigot who, following Pope, sees the most good in striving to see least ill, and who examines his own canons in the light of such common sense as we venture to hope is given to every medical student who aspires to be a doctor.

There is in modern medicine a vast amount of that filth and superfluity of sin of which St. James speaks. We postulate on a grand scale in comparison with Galen, Avicenna, Copho, or that arch-postulant who discovered the lacteals. But it is free to everyone to question the accuracy of lines of treatment founded on postulates, and it is no defence, in logic, to be fallacious in conclusions because your opponent happened to be wrong in a middle clause. Every day we are quietly discarding some of the rubbish we have patiently gathered, for it is only by such rejection, such periodical cleanings out of the lumber-room of science, that any advance is made possible. It is true enough that he would be a very unwise student who would dare argue the value of the specific theory of disease with an examiner; but that does not prevent him from arguing this and any other point with his teacher. And let me add that I know of no teacher in any reputable school of medicine who will not welcome such inquiry and do his utmost to reason with heresy where he meets it. The unfortunate part of the business is that few students demand proofs; the majority are content with experiments, assertions, statistics, and the whole gamut of false struts with which unwholesome theories are propped up in ponderous text-books. There is a story told of Pinel, the talented author of the *Nosographie Philosophique*, and in his day, before the advent of Bichat, the great authority on synovial membranes, who, when interrupted in one of his assertions by a student with a pertinent but impudent question, at once stopped with the remark: "Good lad, always ask why," and do not be satisfied if you are told, as I tell you now, that nobody knows why!"

"There exists a disinclination to tackle big problems," wrote the critic Vosmaer, "and to follow them to their logical conclusions, no matter whither these lead, a fear to question and reject—and we call that politics." He might have substituted "medicine," for the spirit of destructive criticism, so far as essentials and not minor issues are concerned, appears to be dying out. Already it demands an amount of transcendental heroism to impugn the efficacy of Preparation 606! And two years ago none but a professional Luther would have dared to attack opsonins or the accepted theories of nerve regeneration!

I have already said that I regard the safeguarding factors as being three in number—toleration, logical reasoning, and a sense of humour. But there remains a fourth—the study of the history of medicine. It is a study too profoundly neglected by medical men and students, and yet it is one which will help us very much in realising the folly of systematising, classifying, and theorising on postulates, for it teaches above all the truth of Leicester's warning to Elizabeth: *Nicht Stimmenmehrheit ist des Rechtes Probe!*

To plagiarise for a moment Mrs. Besant, all our professional troubles arise from thinking of our science as a separate unit, and then revolving on our own mental axes, thinking only of our separate interests and aims. The study of the history of science, and of medical science in particular, will counteract to a large extent that tendency, for it will teach us that truth is mathematical, and, as Emerson expresses it, without casualty, without eccentricity in all its vast and flowing curve. Once that truth is fully realised the heresy that asks why and refuses to be bound by an authority of to-day, liable to be overthrown to-morrow by statistics that do not need Professor Pearson's acumen to be demonstrated unreliable, or by dicta which are flatulent commonplaces at their best and absurd negations of knowledge at their worst, becomes not merely excusable but absolutely necessary.

